# False-Negative Initial Magnetic Resonance Imaging in Acute Posterior Circulation Stroke: A Case Report Describing Locked-in Syndrome

**DOI:** 10.7759/cureus.11352

**Published:** 2020-11-05

**Authors:** Christian L Castaneda, Jee Ah (Christina) Rhee, Joon Ha H Woo, Michael P Lerario

**Affiliations:** 1 Division of Pulmonary and Critical Care Medicine, New York-Presbyterian Queens, Flushing, USA; 2 Department of Internal Medicine, New York-Presbyterian Queens, Flushing, USA; 3 Department of Medicine, New York-Presbyterian Queens, Bayside, USA; 4 Department of Neurology, Weill Cornell Medicine, New York, USA; 5 Department of Neurology, New York-Presbyterian Queens, Flushing, USA

**Keywords:** magnetic resonance imaging (mri), neurocritical care, posterior circulation, stroke

## Abstract

Locked-in syndrome is defined as quadriplegia and anarthria with the preservation of consciousness. Typically, locked-in syndrome is caused by an insult to the ventral pons secondary to trauma or vascular disease. Presented herein is a case of a locked-in syndrome with an initial MRI with no restricted diffusion and clinical deterioration over the course of four days. Repeat interval MRI demonstrated bilateral pontine ischemia.

## Introduction

As the identification of acute ischemic stroke is greatly dependent on neuroimaging, negative computed tomography (CT) and magnetic resonance imaging (MRI) in the context of focal neurological deficits demand heightened awareness of technology limitations and the need for follow-up imaging. It has been described in the literature that posterior circulation ischemic strokes may not be readily seen on MRI for up to 24 hours. However, with the increasing ease of access to MRI, many patients are receiving scans within 24 hours of presentation. Herein, we present the case of a patient with locked-in syndrome whose initial presentation was bilateral lower extremity weakness and dysarthria with an initial MRI with no restricted diffusion noted.

## Case presentation

The patient was a 49-year-old male who presented after two days of slowly progressive dysarthria and subjective feeling of bilateral lower extremity weakness, which resulted in a fall on the day of admission. His past medical history was significant for lymphoma in remission since 2010, hypertension, diabetes mellitus, congestive heart failure, coronary artery disease with percutaneous coronary intervention, and coronary artery bypass graft. The patient denied any dizziness, diplopia, upper extremity weakness, or shortness of breath. On physical examination, cranial nerves (CN) were intact, including CN IX through XII, despite his mild dysarthria. The motor examination showed 5/5 strength in all extremities but there was a positive motor drift in the bilateral lower extremities. Cerebellar signs were negative, and sensory testing was intact bilaterally in the upper and lower extremities. The patient was admitted for a stroke workup with a National Institutes of Health Stroke Scale (NIHSS) of 5 (based on dysarthria, language, and motor drift in the bilateral lower extremities).

**Figure 1:**
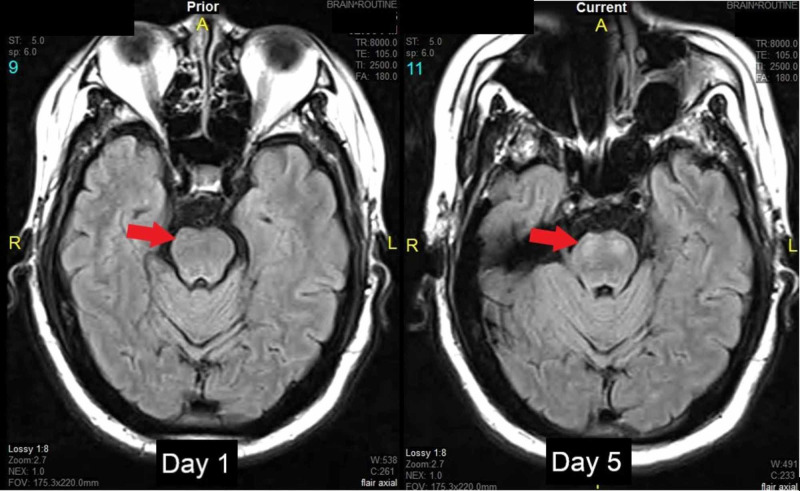
MRI slice of the pons on admission vs Day 5 Displayed are fluid-attenuated inversion recovery (FLAIR) sequences showing no acute pathology initially. Follow-up magnetic resonance imaging displayed bilateral basilar pontine infarcts not previously seen. These findings correlated with the patient’s clinical deterioration.

Initial CT and MRI scans of the brain showed no acute pathology, with only a chronic right corona radiata infarct. CT angiography showed a severe narrowing in the left vertebral artery and a moderate narrowing of the right M1 middle cerebral artery. Over the course of the next four days, the patient developed significantly worsening quadriparesis, most notably in the upper extremities with progressive dysphagia and diplopia. Repeat MRI on Day 5 of his hospitalization showed bilateral basilar pontine infarcts on fluid-attenuated inversion recovery (FLAIR) sequences (Figure 1). The NIHSS progressed to 12 on Day 5, and the patient required intubation for airway protection. Physical exam at this time had progressed to quadriplegia and complete anarthria with preservation of eye movement. After tracheostomy, percutaneous enteral access, and an extended period in the critical care unit, the patient was discharged to a long-term rehabilitation center.

## Discussion

With the increase in stroke centers nationwide, more patients are undergoing MRI scanning within the first several hours of presentation to the hospital [1]. Imaging studies on presentation, including CT, MRI, and vascular studies, localizes the foci of infarction in the majority of patients [2-3]. While diffusion-weighted imaging (DWI) has become synonymous with the acute inpatient management of stroke, reported sensitivity in ischemic stroke ranges from 90% to 98% [3].

False-negative diffusion-weighted imaging is especially prevalent in patients with posterior circulation and lacunar strokes [4-7]. One study in patients presenting with acute vestibular symptoms describes false-negative MRI scans in up to 53% of small strokes (< 10 mm) and 7.8% of large strokes (> 10 mm) [8]. In fact, one 2014 study described a 9.48% false-negative DWI rate with a mean time from symptom onset to MRI of 4.3 hours [9]. Another study specifically examining patients with clinically diagnosed ischemic stroke found that 29% of patients had no DWI lesion [10]. These authors hypothesized that the ability to visualize an acute ischemic lesion on DWI was directly related to the amount of cellular swelling and, therefore, the duration of ischemia [10]. Because natural infarct evolution is to be expected, future studies involving more advanced MRI techniques and repeat neuroimaging after 72 hours may provide more insight into the diagnosis and progression of ischemic strokes. 

There is some evidence to suggest that structured clinical examinations, such as the HINTS test (head impulse, nystagmus, test of skew), are more sensitive and specific than early MRI in acute posterior circulation ischemic stroke. HINTS test is a three-part oculomotor test that differentiates central vertigo from peripheral vertigo with 99% sensitivity and 97% specificity in patients presenting with the acute vestibular syndrome. In this case, initial negative imaging findings obfuscated the diagnosis despite the physical findings. It was only after clinical deterioration and repeat neuroimaging that the diagnosis of posterior circulation stroke was made.

## Conclusions

Evidence suggests that in patients presenting with symptoms consistent with acute posterior circulation stroke, the sensitivity of neuroimaging within the first 24 hours may be substantially lower than that of clinical examination. Physicians should be aware of this limitation, perform thorough and specific neurological examinations, and follow-up with sequential imaging studies for early detection of natural infarct evolution.
